# Neonatal Screening for CAH in Sweden—Results of Implementing Second-Tier Testing

**DOI:** 10.3390/ijns12020029

**Published:** 2026-05-01

**Authors:** Karin Engström, Rolf H. Zetterström, Anna Wedell, Anna Nordenström

**Affiliations:** 1 Centre for Inherited Metabolic Diseases, Karolinska University Hospital, SE-171 76 Stockholm, Sweden; karin.m.engstrom@regionstockholm.se (K.E.); rolf.zetterstrom@ki.se (R.H.Z.); anna.wedell@ki.se (A.W.); 2 Department of Molecular Medicine and Surgery, Karolinska Institutet, SE-171 76 Stockholm, Sweden; 3 Department of Women’s and Children’s Health, Karolinska Institutet, SE-171 76 Stockholm, Sweden; 4 Pediatric Endocrinology Unit, Astrid Lindgren’s Children’s Hospital, Karolinska University Hospital, SE-171 76 Stockholm, Sweden

**Keywords:** neonatal screening, congenital adrenal hyperplasia, CAH, 21-hydroxylase deficiency, CYP21A2, second-tier testing, liquid chromatography–tandem mass spectrometry, dried blood spots, DBS, positive predictive value

## Abstract

Newborn screening for congenital adrenal hyperplasia (CAH) is effective in identifying patients with severe forms before a potentially lethal crisis, but has a relatively high false-positive rate. The aim of this study was to improve the national neonatal screening program in Sweden and the positive predictive value by implementing LC-MS/MS second-tier testing. A combination of two independent parameters, the steroid hormone ratio (androstenedione+17-hydroxyprogesterone)/cortisol and the concentration of 21-deoxycortisol and adjustment of cut-off levels resulted in an increase in the positive predictive value (PPV) from 14% to 84% for full-term infants. In total, the false-positive screening cases decreased by 88%. *CYP21A2* genotyping was used to determine the severity of CAH in identified cases. We report on the stepwise approach that was used to optimize the cut-off levels for full-term and preterm infants in order not to miss any true cases in the process.

## 1. Introduction

Congenital adrenal hyperplasia (CAH) due to 21-hydroxylase deficiency affects about 1/10,000–1/20,000 newborn babies worldwide, and 1/11,000 in Sweden [1,2,3]. It is an autosomal recessive disease caused by pathogenic variants in the *CYP21A2* gene (OMIM 613815), causing impaired or abolished enzyme activity resulting in varying degrees of cortisol and aldosterone deficiency. The resulting increased ACTH production causes accumulation of precursors such as 17-hydroxyprogesterone (17-OHP) and androgen excess [1,4,5]. In the severe salt wasting (SW) form, this may lead to life-threatening adrenal crisis with hyperkalemia and hyponatremia in the neonatal period, which may be lethal if untreated. Individuals with the less severe simple virilizing (SV) form have enough residual enzyme activity to maintain electrolyte and water balance under normal conditions and considerably less risk of developing an adrenal crisis. In both these forms, the prenatal androgen excess results in varying degrees of virilization of the external genitalia in 46,XX fetuses, with clitoral enlargement and formation of a sinus urogenitale. The SW and SV forms of CAH constitute classic CAH. In the least severe, non-classic (NC), late onset, form, there are no signs of prenatal virilization but due to the relative lack of cortisol and less negative feedback on the pituitary, signs of androgen excess develop over time. The androgen excess may lead to increased growth velocity and bone age with premature closure of the epiphysis, resulting in short adult stature, increased androgen symptoms, and precocious pseudo puberty, menstrual disturbances, and subfertility [6].

Neonatal screening for CAH, using 17-OHP as a marker for disease, was first started in the 1970’s with the aim to prevent salt crisis and death in the neonatal period [7]. Since then, more than 40 countries and all states in the USA have implemented screening programs [5,8]. The Swedish national neonatal screening program started in 1986. Screening has been shown to be successful in preventing salt crises, especially in boys, and shortens the time of uncertainty of sex assignment in girls with CAH [2]. The primary aim of most screening programs is to avoid neonatal salt crisis and death [1,2,8,9,10].

Neonatal screening for CAH has been problematic in that the false-positive rate has been relatively high, especially among preterm infants [3,11]. With the aim of improving outcomes, cut-off levels for 17-OHP adjusted for birth weight and gestational age have been implemented [12,13,14]. Despite these efforts, the screening for CAH has had the highest false-positive rate among the screening disorders, with positive predictive values (PPVs) ranging from less than 1% to 30% [15].

A 26-year follow-up of the Swedish screening program, using an immunoassay for 17-OHP, was performed [15]. The sensitivity for the different severities of the disease, as defined by the *CYP21A2* genotype, showed that the sensitivity for SW forms was 100%, for SV, 80%, and for the NC form with no risk of developing salt crisis, 32%. The overall PPV was 13.4%, higher for full-term infants, at 25.1%, and for preterm infants, as low as 1.4%. The referral rate was less than 0.06%.

Radio immunoassay and later DELFIA methods have been used in most screening programs [8]. The development of tandem mass spectrometry techniques in combination with liquid chromatography (LC-MS/MS) has opened new possibilities for improvements in NBS. Specific measurements of several adrenal steroids in dried blood spots (DBS) are now possible. Androstenedione (A4), 17-OHP, and cortisol (F) were measured as a second-tier test when the initial 17-OHP was elevated [16]. The (17-OHP + A4)/F ratio discriminates sick from healthy better than 17-OHP alone, but if set with too high a cut-off and used as the only parameter, babies with the salt-wasting form were missed in the screening [17,18]. A sensitive and possibly more specific marker for 21-hydroxylase deficiency is 21-deoxycortisol [19,20]. It has, however, been shown not to be useful as the sole marker in the screening [21].

Several strategies have been developed using individual steroid measurements and combinations of ratios to improve specificity without loss in sensitivity [22,23].

The aim of this study was to improve the specificity and positive predictive value for both full-term and preterm infants in the Swedish national neonatal screening program by implementing a second-tier test based on a combination of a steroid ratio and the concentration of 21-deoxycortisol as individual parameters. In Sweden, the diagnosis is confirmed with *CYP21A2* genotyping. The genotype could, therefore, be used to determine the severity of identified cases in the screening. A stepwise process was used to determine the cut-off levels for full-term and preterm infants and the effects on the referral rate and PPV in order not to miss any true cases in the process.

## 2. Materials and Methods

### 2.1. The Strategy

The first-tier analysis of 17-OHP using an immunoassay was identical to the setup used in the screening previously [15]. A fast second-tier analysis using LC-MS/MS to determine 17-OHP, F, A4, 11-deoxycortisol, and 21-deoxycortisol was developed. The second-tier assay was evaluated based on the ratio calculated from (17-OHP + A4)/F, as well as the concentration of 21-deoxycortisol as two independent parameters. The screening filter paper cards of known patients with CAH and of false-positive cases previously identified were retrospectively analyzed using the second-tier analysis. During a 6-month test period, the second-tier LC-MS/MS analysis was performed in parallel with the first-tier screening test.

The cut-off levels for preterm infants were assessed with separate approaches for those with gestational age (GA) < 34 weeks and those with GA 35–36 weeks.

The main outcome measure was the sensitivity of the screening for salt-wasting CAH. The most important secondary outcome measures were the PPV (positive predictive value) and referral rates for full-term and preterm infants, as well as sensitivity for milder forms of CAH. Throughout the process, we took advantage of the good genotype/phenotype correlation. We also assessed whether the level of 21-deoxycortisol and/or the ratio can predict disease severity.

### 2.2. Sampling

Sweden has a population of 10 million, and about 100,000 babies are born annually. There is one national screening laboratory, and virtually all newborn babies participate in the newborn screening program [15].

The families are given written and oral information at the time of sampling, and an opt-out procedure is employed. DBS samples are collected as soon as possible after 48 h from birth (on Revvity 226 Ahlstrom paper (Revvity, Greenville, SC, USA)) and sent to the national screening laboratory.

After the screening is completed, the samples are stored at 4 °C in the national Swedish PKU-biobank. For evaluation of the new screening method, samples of newborns with confirmed CAH, together with samples from newborns with a false-positive CAH screening result and samples with a normal screening result, were retrieved from the PKU-biobank. All five steroid hormones included in this study have shown retained concentrations in DBS after 1 year of storage at 4 °C [24].

### 2.3. Materials

Acetonitrile, methanol (gradient grade for LC), and formic acid (for analysis) were purchased from Merck (Darmstadt, Germany). The 2-propanol of LC-MS grade was from Honeywell (Seelze, Germany). Ammonium acetate was purchased from Sigma-Aldrich (Merck, Darmstadt, Germany). Standards and controls in DBS containing cortisol, 21-deoxycortisol, 11-deoxycortisol, 17-hydroxyprogesterone, Δ4-androstenedione, and 21-hydroxyprogesterone used for the second-tier assay were purchased from Lab Systems (Vantaa, Finland).

The isotope-labeled standards cortisol-d4, 21-deoxycortisol-d8, 11-deoxycortisol-d5, and 17α-hydroxyprogesterone-d8 in methanol, and d7-Δ4-androstenedione-13C3 in acetonitrile were obtained from Cerilliant (Round Rock, TX, USA). A fresh solution of internal standard was prepared weekly at a concentration of 20 nmol/L in methanol/water (70/30 *v*/*v*).

### 2.4. First-Tier Immunoassay

The concentration of 17-OHP was measured in all specimens by time-resolved fluoroimmunoassay using GSP^®^ instruments (Revvity, Turku, Finland) according to the manufacturer’s protocol. The results of this first-tier CAH screening were interpreted in relation to the gestational age (GA). All samples with a concentration above the cut-off were re-run with the first-tier assay and simultaneously analyzed to validate that the initial screening result was correct, with no mix-up of samples and with the second-tier LC-MS/MS method.

### 2.5. Second-Tier LC-MS/MS Method

One 3.2 mm blood spot was punched out of each DBS calibrator, control, and sample into a 96-well plate, and 80 μL of internal standard mix (20 nmol/L) was added. The plate was incubated at room temperature for 30–60 min in an orbital shaker at 1000 min^−1^. Thereafter, 30 μL of water was added to each well. After mixing, the supernatants were transferred to vials for analysis.

LC-MS/MS was performed using an Acquity UPLC connected to a Xevo TQS mass spectrometer equipped with a unispray ion source (both Waters, Milford, MA, USA). Separation was achieved with an Acquity HSS T3 column (1.7 μm, 50 × 2.1 mm) (Waters) at 60 °C and a flow rate of 0.8 mL/min. Mobile phase A consisted of a 2 mM ammonium acetate/0.1% formic acid solution in water, while mobile phase B was methanol with 2 mM ammonium acetate/0.1% formic acid. The injection volume was 10 μL, and the separation used the following gradient. First, 50% of mobile phase B was kept for 1 min, followed by a linear gradient to 75% mobile phase B from 1 to 1.75 min, then 75–98% mobile phase B (linearly) from 1.75 to 2 min, which was maintained for 0.25 min. Thereafter, the column was re-equilibrated with the initial conditions for 0.25 min. Total runtime was 2.5 min. Positive ionization was used, and the settings for the mass spectrometer are given in Supplementary Information, Tables S1 and S2.

### 2.6. Genotype

The patients were divided into genotype groups based on the least severe *CYP21A2* allele. Homozygosity for null alleles with no residual enzyme activity is associated with the most severe SW phenotype group A; the I2 splice variant (c.293-13C>G) with less than 1% enzyme activity usually results in the slightly less severe but SW form group B. p.Ile173Asn, (c.518T>A) typically results in SV (group C), while p.Arg342Trp (c.1024C>T) and p.Glu141Lys (c.421G>A) (group D), as well as p.Val282Leu (c.844G>T) (group E), are associated with NC CAH [4,25,26,27,28]. For patients with other variants, the published enzyme activity was used to classify the individual into a genotype group.

### 2.7. Statistics

Microsoft Excel (Microsoft, Redmond, WA, USA) was used for data management and statistical analysis. Graphics were created using RStudio Statistical Software (v2025.09.0; Posit Software, PBC, Boston, MA, USA 2025) or Microsoft Excel.

## 3. Results

### 3.1. LC-MS/MS Method

**Figure 1:**
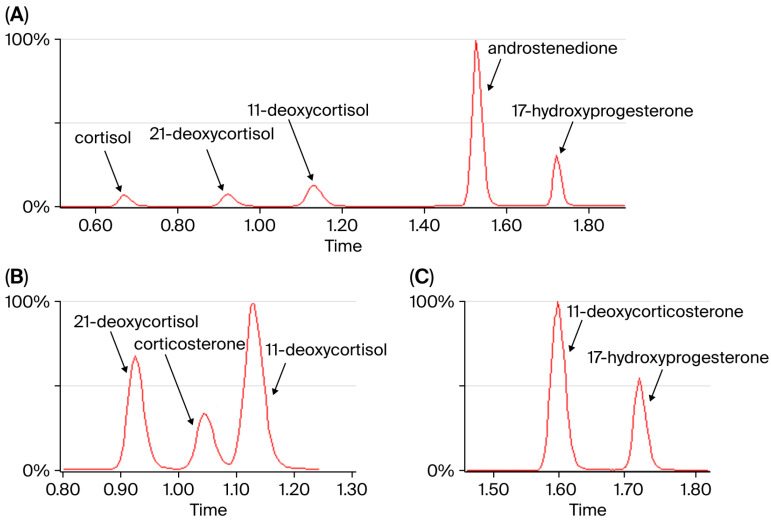
(**A**) Chromatogram with all five steroids. The runtime per sample is 2.5 min. (**B**) Separation of isobaric compounds 21-deoxycortisol, corticosterone, and 11-deoxycortisol, *m*/*z* 347.2 → 311.1. (**C**) Separation of isobaric 17-hydroxyprogesterone from 11-deoxycorticosterone, *m*/*z* 331.2 → 109.1.

We developed a fast LC-MS/MS method for the quantification of five steroid hormones in DBS. The sample preparation is simple: an extraction followed by a dilution. No time-consuming evaporation or reconstitution step is needed. The LC method separates the analytes well, and the analysis time is short (2.5 min per sample). The chromatogram with the five steroids analyzed in this method is shown in Figure 1. The method separates the isobaric analytes 21-deoxycortisol and 11-deoxycortisol from endogenous corticosterone (Figure 1B). 17-OHP is separated from isobaric 11-deoxycorticosterone (Figure 1C). Further specifications on the method’s performance are given in the Supplementary Information, Table S3.

### 3.2. Retrospective Analysis of True-Positive and False-Positive CAH Cases and Establishment of Cut-Off Values

To establish cut-off values for the second-tier method, all confirmed CAH cases identified by newborn screening between 2015 and 2020 were analyzed retrospectively, with a total of 49 samples. It was especially interesting and important to compare the confirmed CAH samples with samples from newborns that previously had a false-positive outcome in the CAH screening, since these were the samples proven to be most difficult to distinguish from the true-positive samples in the past. Newborn screening samples with false-positive results for CAH in the first-tier immunoassay (*n* = 155) were therefore analyzed with the second-tier method, in addition to several random healthy newborns (*n* = 46).

**Figure 2:**
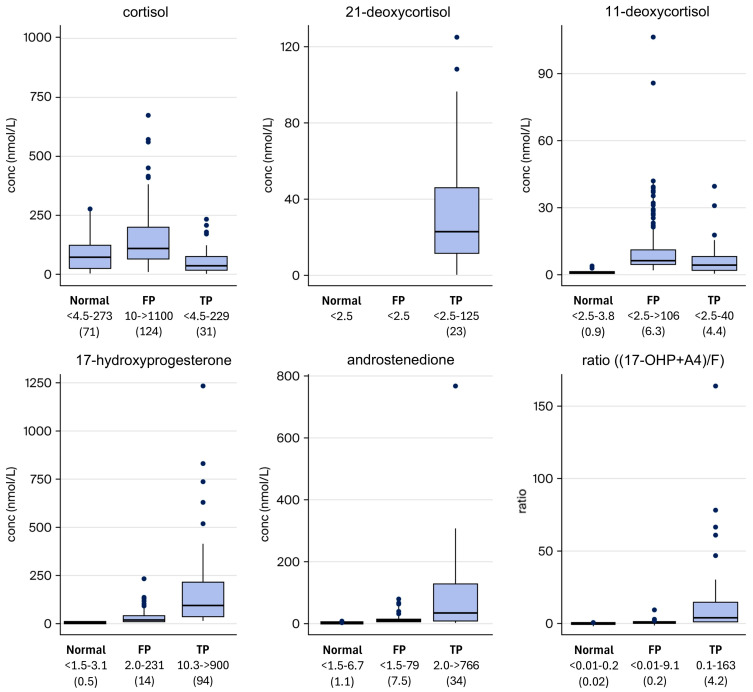
Concentration of the measured analytes in normal (healthy) newborns, false-positive (FP) newborns, and true-positive (TP) newborns. The concentration range for each group (nmol/L) is given in the figure with the median in parentheses.

A comparison between newborns with a normal screening result, a false-positive CAH result, and confirmed CAH cases is visualized in Figure 2. All three groups consist of a mixture of full-term and preterm newborns.

None of the analytes alone could successfully distinguish between healthy newborns and CAH in a satisfying way. A concentration of 21-deoxycortisol above the limit of quantification (2.5 nmol/L) was seen in 42 out of 49 (86%) of the newborns with CAH. In addition, 21-deoxycortisol was not found in any false-positive screening cases, nor in healthy newborns. This indicates that 21-deoxycortisol is a good indicator of disease. However, not all newborns with CAH have a measurable concentration of 21-deoxycortisol, which is why another parameter is required to identify them in the screening.

17-OHP and androstenedione were increased in newborns with CAH, while cortisol in many cases was low, possibly making the ratio a more sensitive indicator than the concentration of 17-OHP by itself. However, some of the confirmed newborns with CAH had a normal ratio. For those that lacked 21-deoxycortisol, the ratio (17-OHP + A4)/F ranged from 1.0 to 160.

**Figure 3:**
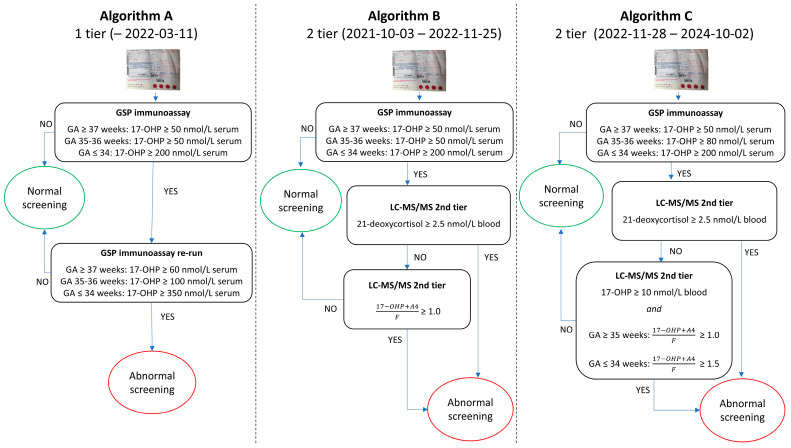
The first-tier approach (Algorithm A) was used prior to the introduction of the second-tier method and during the initial test period. The initial second-tier algorithm (Algorithm B) was derived from the results of the retrospective study. During the test period, it was used in parallel with Algorithm A. The final (Algorithm C) was proposed after evaluation of data from one year of second-tier testing. GA = gestational age.

The screening algorithm with a 2-tier protocol was, therefore, based on either a concentration of 21-deoxycortisol above the limit of quantification (2.5 nmol/L) or an elevated ratio (17-OHP + A4)/F (Figure 3, Algorithm B).

### 3.3. Evaluation of Cut-Offs After One Year with Second-Tier Testing

After one year of second-tier testing (pilot study included), the screening algorithm was re-evaluated. The second-tier approach (B) was compared with the first-tier approach (A), and the number of referrals decreased from 88 to 31 cases (including 6 true-positive CAH cases).

#### 3.3.1. Full-Term Infants

Most false-positive cases among full-term infants were eliminated with Algorithm B. Despite a low concentration of 17-OHP, some of the remaining false-positive results had a ratio above the cut-off due to a markedly low cortisol level. The criterion that the concentration of 17-OHP had to be above 10 nmol/L for a referral was therefore included.

#### 3.3.2. Gestational Age 35–36 Weeks

The number of referrals at GA 35–36 weeks was reduced from 23 (Algorithm A) to 9 (Algorithm B). Out of 482 samples analyzed with the second-tier method, nearly half (215, 45%) were from infants born at GA 35–36 weeks. Since less than 4% of all infants are born at GA 35–36 weeks, this was a huge over-representation.

We analyzed samples from all patients with CAH born at GA 35 or 36 weeks from the start of the screening in 1986 (Supplementary Material). All newborns with classic CAH born at GA 35–36 weeks had a first-tier 17-OHP above 100 nmol/L serum. Two patients with non-classic CAH had a lower 17-OHP; however, they had neither a ratio above 1.0 nor a measurable concentration of 21-deoxycortisol and would, therefore, not have been referred if they had been analyzed with the second-tier method.

The ratio in the second-tier assay was compared to the 17-OHP concentration from the first-tier immunoassay (Supplementary Figure S1). Surprisingly, most of the samples with a first-tier 17-OHP above 100 nmol/L serum had a ratio far below 1.0. On the contrary, the samples with a 17-OHP in the range 50–80 nmol/L serum accounted for most of the referrals based on the second-tier algorithm. Increasing the first-tier cut-off to 80 nmol/L serum, therefore, reduced the number of second-tier runs and the number of referrals drastically.

#### 3.3.3. Gestational Age ≤ 34 Weeks

For the preterm infants born at gestational age (GA) 34 weeks or earlier, the number of false-positives did not decrease with the new second-tier algorithm (Algorithm B). Therefore, we analyzed more preterm samples to see if a different cut-off could be used for this group. Retrospective second-tier analysis was performed on samples from all preterm infants born in Sweden since 1986 at GA ≤ 34 weeks with a CAH diagnosis, in total 9 samples. All samples had measurable 21-deoxycortisol (>2.5 nmol/L) and were, therefore, considered positive regardless of the (17-OHP + A4)/F ratio. In addition, all samples from newborns with classical CAH had a ratio above 1.5, except two samples from newborns who had received treatment with hydrocortisone prior to screening. Based on these results, we decided to use a cut-off ratio of 1.5 for those born at or before GA 34 weeks. Patients with more severe forms of CAH (SW) had ratios above that, but less severe (SV and NC) forms are potentially at risk of being missed by the higher ratio. However, they will likely be identified due to the presence of 21-deoxycortisol. In return, the number of false-positive cases will be drastically reduced.

### 3.4. Results of the First Three Years with Second-Tier Testing

**Figure 4:**
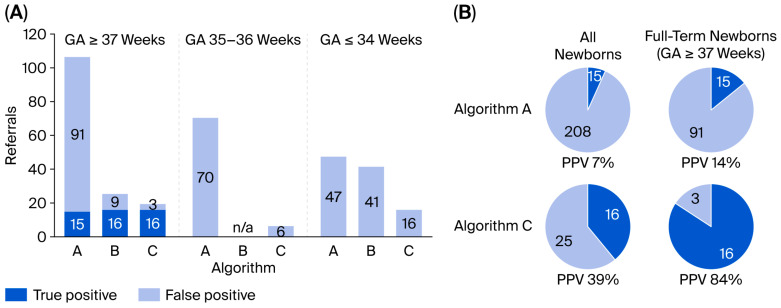
(**A**) Number of referrals with the three different algorithms (Algorithm A–C). No data are available for Algorithm B for GA 35–36 weeks since the first-tier cut-off was changed during the period, and samples with an initial 17-OHP of 50–79 nmol/L serum did not undergo the second-tier test. (**B**) Evaluation of screening Algorithm A and C.

The three different screening Algorithms (A–C) that were used were compared based on the data from the first three-year period with the second-tier method (Figure 4). With Algorithm C, false-positive screening outcomes were almost eliminated in term babies, and in total, false-positive screening cases decreased by 88%. The total positive predictive value increased from 7% to 39% with this second-tier approach.

### 3.5. Biochemistry—Genotype Correlation

**Figure 5:**
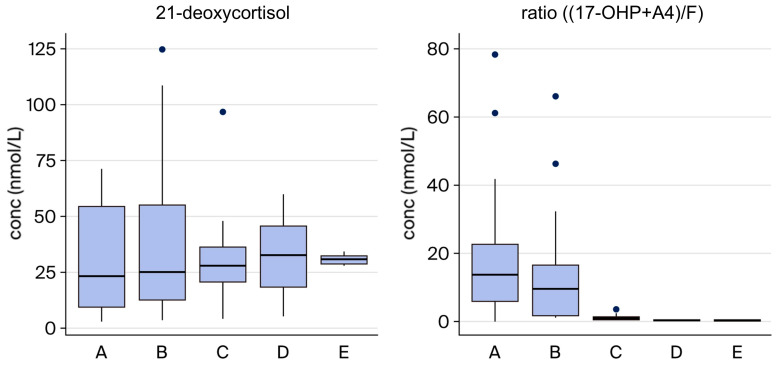
Correlation between severity of disease and biochemistry. Newborns with the most severe salt-wasting form of CAH (groups A–B), the simple virilizing form (group C), and non-classic CAH (groups D–E) are compared.

Patients with CAH can be divided into different genotype groups based on the severity of the disease based on their *CYP21A2* genotype. To assess if the severity of the disease could be predicted from the second-tier results, the concentrations of 21-deoxycortisol and the ratio (17-OHP + A4)/F were compared between the different genotype groups (Figure 5). The concentration of 21-deoxycortisol in the samples did not show a clear correlation to the severity, while the ratio was generally higher in samples from newborns with the salt-wasting form (groups A–B).

## 4. Discussion

This study shows that the neonatal screening for CAH was clearly improved using a second-tier testing approach adjusted for and related to gestational age. We used a first-tier immunoassay followed by a second-tier LC-MS/MS analysis. A ratio of (17-OHP + A4)/F was calculated. Using the ratio and the detection of 21-deoxycortisol as two independent measures, independently qualifying for referral, the overall PPV was increased from 7 to 39%. The PPV for term newborns increased from 14% to 84%. Among the preterm infants, the number of false-positives decreased by 65%.

This new algorithm for neonatal screening for CAH was established using a stepwise approach for the implementation to ensure high sensitivity and avoid false negatives.

By adjusting the cut-offs for the first-tier 17-OHP measurement and the cut-off level for the ratio in the second-tier test for preterm infants GA ≤ 34 weeks, the false positives were drastically reduced.

In the three-year follow-up with the new algorithm, we have not identified any missed cases. However, not enough time has passed to be able to rule out missed cases. For a proper follow-up of a neonatal screening efficiency, more than a decade is required. The primary aim of the screening is not to identify individuals with the NC form of CAH. However, when an infant with an elevated screening result is found to have NC CAH screening, the clinical situation and the treating physician will decide if and when the child should be treated. We do not consider these to be false-positive cases. The fact that the child is identified may be an indication that the steroid metabolism for this individual results in somewhat elevated androgen levels and possibly the development of premature adrenarche.

The second-tier ratio has been shown to be effective in improving the PPV of CAH screening but may have a few weak aspects. The ratio was shown to have inherent difficulties if set with a too-high cut-off and used as the only parameter, when 25% of babies with the salt-wasting form were missed in the screening [17]. Increased ACTH and stress may result in elevated cortisol and, therefore, a lower ratio. In addition, the 17OHP level increases over time after birth in patients with CAH, which makes the time of sampling crucial. In recent years, some neonatal intensive care units treat extremely preterm babies with hydrocortisone in modest doses during the first 10 days of life, intending to prevent bronchopulmonary dysplasia [29,30]. It is not known to what extent this may affect the screening outcome of these infants.

In our study, virtually all patients with CAH had measurable 21-deoxycortisol. During the three years of this study, all true-positive samples had a concentration of 21-deoxycortisol above the limit of quantitation of the method used (2.5 nmol/L). The samples from the retrospective study, however, showed that this metabolite is not present in all samples from newborns with CAH. If this is due to concentrations below detection or because 21-deoxycortisol is not stable over time in filter paper samples stored for years at 4 degrees centigrade is not known; the stability of the steroid hormones has not been confirmed for as long as the retrospective samples were stored prior to analysis. While some reports suggest that 21-deoxycortisol is present in all newborns with CAH, others report results like ours [21].

A recent study reported that plasma 21-deoxycortisone is an even better marker than 21-deoxycortisol for CAH, and is present in higher concentrations in the samples [31]. This seems to be the case in DBS from newborns as well, and it might be a superior marker for CAH [32].

The strengths of this study are that the change in procedure was performed in a stepwise fashion and in parallel with the previous screening. The results for individual cases were evaluated in relation to disease severity using the *CYP21A2* genotype. The 3-year follow-up period is, however, too short to rule out the possibility of missed cases with classical CAH, SW, and SV phenotypes.

## 5. Conclusions

This two-tiered screening algorithm for CAH has greatly improved our national neonatal screening. The false-positive screening cases decreased by 88%, the overall PPV increased from 7 to 39%, and in full-term infants, from 14 to 84%. Among term newborns, the false-positive referrals were almost eliminated. 21-Deoxycortisol proved to be a useful marker with high specificity. In our study, no healthy newborns had a measurable concentration of 21-deoxycortisol. The steroid ratio (17-OHP + A4)/F, used in many screening programs, has a lower specificity. We found it effective to combine the two independent LC-MS/MS measurements: the ratio and 21-deoxycortisol.

## Data Availability

The raw data supporting the conclusions of this article will be made available by the authors on request.

## Supplementary Materials

The following supporting information can be downloaded at: https://www.mdpi.com/article/10.3390/ijns12020029/s1, Table S1: MS/MS instrument parameters for the second-tier LC-MS/MS method. Table S2: Parameters for detection of the different steroids and internal standards. Table S3: Method performance. Figure S1: Second-tier (17-OHP + A4)/F ratio plotted against the 17-OHP concentration from the first-tier immunoassay.

## Abbreviations

The following abbreviations are used in this manuscript:

CAH	Congenital adrenal hyperplasia
SW	Salt wasting
SV	Simple virilizing
NC	Non-classic
F	Cortisol
17-OHP	17-hydroxyprogesterone
A4	Androstenedione
GA	Gestational age
PPV	Positive predictive value
LC-MS/MS	Liquid chromatography tandem mass spectrometry
DBS	Dried blood spot
FP	False-positive
TP	True-positive
