# Supplementary material for: Neonatal Screening for CAH in Sweden—Results of Implementing Second-Tier Testing
Source: Int J Neonatal Screen. 2026 May 1;12(2):29. doi: 10.3390/ijns12020029 (PMC13214752; doi:10.3390/ijns12020029)
Supplement: Supplementary file 1 [file IJNS-12-00029-s001.zip › IJNS-4211336-supplementary.pdf]

Supplementary

Table S1. MS/MS-instrument parameters for the second tier LC-MS/MS method.

Parameter	
Capillary voltage	1.5 kV
Source temperature	150°C
Dessolvation temperature	650°C
Dessolvation gas flow rate	1000 l/h
Colission gas flow (argon)	0.25 ml/min
Software for system control and data acquisition	MassLynx 4.1 (Waters)

Table S2. Parameters for detection of the different steroids and internal standards.

	**Quantifying transition (Q)***			**Identifying transition (I)****		
**Analyte**	**MRM Transition**	**Cone voltage (V)**	**Collision energy (eV)**	**MRM Transition**	**Cone voltage (V)**	**Collision energy (eV)**
Cortisol	363.1 → 121.1	6	26	363.1 → 97.1	6	30
21-Deoxycortisol	347.2 → 311.1	24	14	347.2 → 121.1	24	25
11-Deoxycortisol	347.2 → 109.0	22	26	347.2 → 97.0	22	26
17-Hydroxyprogesterone	331.2 → 109.1	30	28	331.2 → 97.0	20	22
Androstendione	287.1 → 97.0	26	24	287.1 → 109.0	26	22
Cortisol-d4	367.3 → 121.1	6	22			
21-Deoxycortisol-d8	355.3 → 319.3	18	16			
11-Deoxycortisol-d5	352.4 → 100.0	22	26			
17-Hydroxy-progesterone-d8	339.4 → 100.2	20	22			
Androstendione-13C3	290.2 → 100.0	26	24			

* Steroid identities were confirmed by their retention time as compared to the labeled internal standard and by the identifying transitions (I). **The concentrations were determined using the quantifying transitions (Q) and a linear regression curve.

Table S3. Method performance.

**Parameter**	Intra-assay precision*	Inter-assay precision*	Lower limit of quantitation** (nmol/L)	Upper limit of quantitation*** (nmol/L)	Recovery****
**Cortisol**	5-9%	7-9%	4.5	1100	94%
**21-deoxycortisol**	5-8%	7-9%	2.5	900	91%
**11-deoxycortisol**	6-8%	7-9%	2.5	900	91%
**17-OHP**	6-8%	7-9%	1.5	900	101%
**Androstenedione**	6-7%	7-8%	1.5	1000	97%

*Assay precision was assessed at three different concentrations within the linear range. **The limit of quantification was assessed as the concentration where CV remained 20% or less and S/N <10. ***Method Linearity was not tested above the highest calibrator, but the real upper limit is probably higher. ****Recovery was calculated as the mean of measurement at two different concentrations.

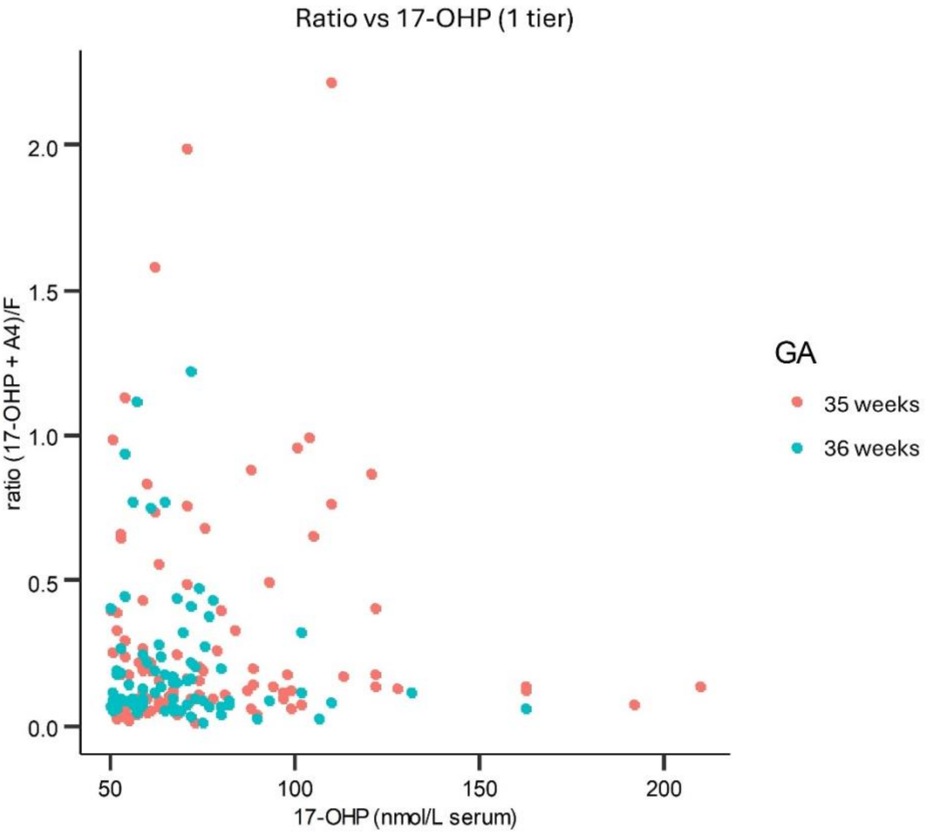

Figure S1. Second-tier (17-OHP+A)/F ratio plotted against the first-tier immunoassay 17-OHP concentration for infants born at gestational age 35-36 weeks. The horizontal line represents the second-tier cutoff of 1.0 (to be exact 0.95 since concentrations above will be rounded off to 1.0). Samples with a 17-OHP concentration in the range 50-80 nmol/L serum (the grey area) accounted for most of the referrals based on the second-tier algorithm.
